# REPRO_PL-Polish Mother and Child Cohort—Exposure, Health Status, and Neurobehavioral Assessments in Adolescents—Design and Cohort Update

**DOI:** 10.3390/ijerph192114167

**Published:** 2022-10-29

**Authors:** Magdalena Janc, Agnieszka Jankowska, Monika Weteska, Agnieszka Brzozowska, Wojciech Hanke, Joanna Jurewicz, Mercè Garí, Kinga Polańska, Joanna Jerzyńska

**Affiliations:** 1 Department of Environmental and Occupational Health Hazards, Nofer Institute of Occupational Medicine (NIOM), 91-348 Lodz, Poland; 2 Department of Pediatrics and Allergy, Copernicus Memorial Hospital, Medical University of Lodz (MUL), 90-329 Lodz, Poland; 3 Department of Environmental Epidemiology, Nofer Institute of Occupational Medicine (NIOM), 91-348 Lodz, Poland; 4 Department of Toxicology, Medical University of Lodz (MUL), 90-151 Lodz, Poland; 5 Department of Environmental Chemistry, Institute of Environmental Assessment and Water Research (IDAEA-CSIC), 08034 Barcelona, Spain

**Keywords:** birth cohort, pregnancy, childhood, adolescence, environmental exposures, health and neurodevelopment

## Abstract

Early life is a crucial window of opportunity to improve health across the life course. The prospective cohort study design is the most adequate to evaluate the longitudinal effects of exposure, the notification of changes in the exposure level and evaluation of the simultaneous impact of various exposures, as well as the assessment of several health effects and trajectories throughout childhood and adolescence. This paper provides an overview of the Polish Mother and Child cohort (REPRO_PL), with particular emphasis on Phase IV of this study. REPRO_PL is conducted in central Europe, where such longitudinal studies are less frequently implemented. In this population-based prospective cohort, which was established in 2007, three phases covering pregnancy (I), early childhood (II), and early school age (III) periods have already been completed. Phase IV gives a uniform opportunity to follow-up children during adolescence in order to evaluate if the consequences of prenatal and early postnatal exposures still persist at the age of 14. Moreover, we will be able to investigate the associations between simultaneous exposures to a broad spectrum of environmental factors, adolescents’ health and neurobehavioral outcomes, and their trajectories within life, which is a novel framework of high scientific, public health and clinical priority.

## 1. Introduction

Adverse exposures during fetal life and the postnatal period influence the physical, emotional and cognitive development of children, and predispose them to an increased risk of various chronic diseases throughout their life course. Socioeconomic, geographical and ethnic differences related to health inequalities from fetal to childhood/adolescence and adulthood life need to be underlined [1,2]. Understanding the stressors that influence the individual, biological, developmental and health trajectory variations related to the onset and evolution of chronic diseases is essential to develop new strategies for optimizing and maximizing the human developmental potential during early life [3]. A better knowledge of such influences requires prospective studies starting from pregnancy, which are represented by birth cohort studies. Over the last 30 years, many birth cohorts have been established in Europe, providing an enormous amount of data and enabling multicohort analyses [1,4,5,6,7,8,9,10]. Cross-cohort collaborations enable meta-analyses of individual participants or pools of data, allow the possibility of identifying smaller effect estimates, and improve the identification of risk groups and factors leading to disease throughout life across countries [1]. This is also crucial for a better understanding and modeling of health trajectories and the causes of disease through the life course [1]. Whereas most of the birth cohorts are conducted in Western Europe (i.e., ALSPAC, INMA, Generation R, EDEN cohorts), Central/Eastern Europe is less frequently represented (i.e., REPRO_PL, Krakow cohort, Slovak PCB study).

The main aim of this paper is to provide an overview of the Polish Mother and Child cohort (REPRO_PL), with particular emphasis on Phase IV of this study, which focuses on a diversity of environmental and lifestyle-related exposures, as well as health and neurobehavioral assessments during the adolescence period. The details regarding the follow-up methodology of the children are crucial for a future cross-cohort collaboration and full understanding of the remaining research questions.

## 2. Objectives

The general aim of the REPRO_PL cohort is to evaluate the impact of occupational, environmental and lifestyle-related exposures during the prenatal period and after birth on pregnancy outcomes and/or offspring health during various stages of life (infancy, childhood and adolescence).

The specific aims refer to:

(a) The evaluation of the main determinants of occupational (when related to mothers), environmental (air pollution and chemicals) and lifestyle-related (diet, tobacco constituents, physical activity and use of new technologies) exposures on pregnancy outcomes (including gestational age, birth weight, birth length and head circumference) and/or offspring health, with special focus on adiposity, cardiometabolic diseases, respiratory/allergic problems and neurobehavioral outcomes.
(b) The evaluation of simultaneous exposures to a broad spectrum of pre- and postnatal occupational/environmental/lifestyle-related factors, including genetic profiling, on health and neurobehavioral outcomes, in an Exposome-based approach.
(c) The evaluation of outcome associations and trajectories throughout childhood and adolescence derived from occupational/environmental/lifestyle-related exposures.

## 3. Material and Methods 

### 3.1. General Description of the Cohort

#### 3.1.1. Study Design and Population

**Figure 1:**
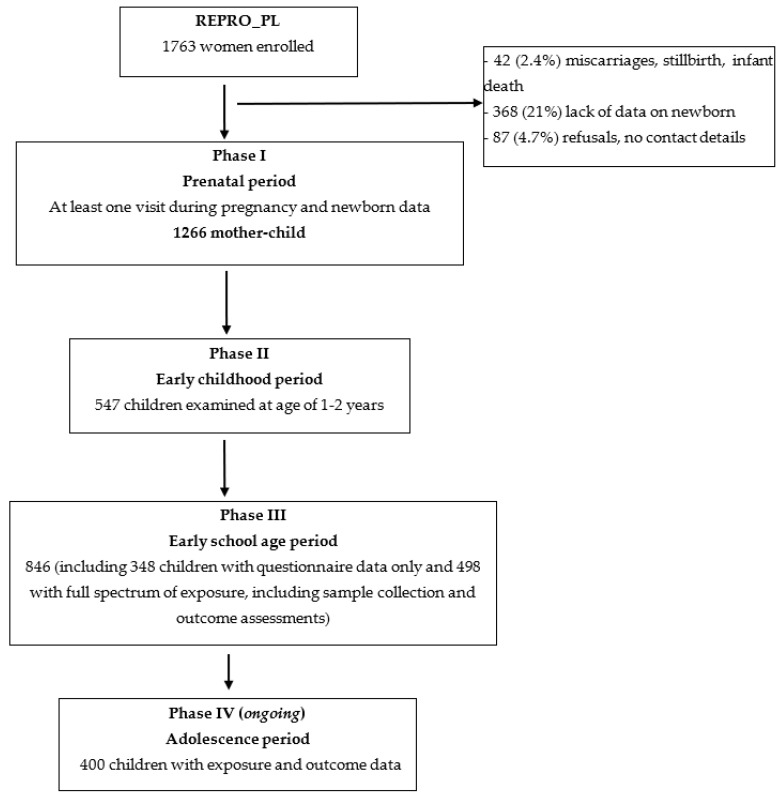
Enrolment and follow-up in the REPRO_PL study.

The Polish Mother and Child Cohort (REPRO_PL) is a population-based multi-center longitudinal study established in 2007 in Poland. The following inclusion criteria were applied: single pregnancy up to the 12th week of gestation, without assisted conception, pregnancy complications and chronic diseases. Figure 1 presents the enrolment and follow-up data of REPRO_PL. The women who agreed to participate in the study were followed-up three times during pregnancy (Phase I of the study, recruitment period: 2007–2011) [11]. The child’s exposure, health status and neurodevelopmental assessments were performed at 1 and 2 years of age (Phase II of the study: 2008–2013) [12] and 7–9 years of age (Phase III of the study: 2014–2019) [13]. Phases I–III of the REPRO_PL cohort have already been completed. The ongoing Phase IV of the study is dedicated to exposure and outcome assessments during the adolescence period (at age of 14).

#### 3.1.2. Response and Follow-Up

A total of 1763 women were recruited in the REPRO_PL cohort (details are provided by [13]), out of which 1266 finished the Phase I study, with at least one visit during pregnancy and newborn data (72%). During Phase II (early childhood, ages 1–2), 718 mothers and their children were invited to participate in the follow-up. Considering financial and organizational possibilities, this phase was limited to a sub-cohort residing in 2 out of 10 regions of Poland, and a total of 547 participants completed it (76%). Phase III (early school age) was implemented into two models: (a) questionnaire data filled in by participants and sent to the study center (completed by 348/602 participants, 58%), and (b) the full spectrum of exposure, including sample collection and outcome assessments by allergologists and psychologists (completed by 498/664 participants, 75%). It is planned that Phase IV (adolescence period) will be completed by 400 participants.

Table 1 presents the general characteristics of the mothers and their offspring who completed Phases I–III of the study. The mean age at childbirth for the women who completed Phase I was 29.2 ± 4.2 years. Similar ages were found for mothers who completed Phases II and III (*p*-value of one-way ANOVA test = 0.1). Concerning maternal education, more than 65% of the women had a university degree, and no differences were noted for each phase of the study (*p*-value of chi-square test = 0.9). About 15% of the women were classified as smokers based on cotinine levels in saliva at the first trimester of pregnancy, and below 10% declared alcohol consumption (no differences were found within each phase of the study; *p*-value > 0.6). A slightly higher birth weight was noted for children who finished Phase III compared to those who completed Phase I or II (*p*-value < 0.05).

#### 3.1.3. Measurements in REPRO_PL

The analyses based solely on the data from the REPRO_PL cohort (Phases I–III) and joint analyses based on the pooled data from the European birth cohorts allowed us to point out a variety of associations between environmental/lifestyle-related factors and children’s health. This multidisciplinary study focuses on several health outcomes, including (1) fetal ultrasound and newborn parameters (biparietal diameter, femur length, gestational age, birth weight and length, and fetal and newborn head and abdominal circumference); (2) child growth parameters within the first 9 years of life (height, weight, skin-fold thickness, waist–hip ratio, and weight/body composition); (3) oral health; (4) infectious diseases and immunity, respiratory health, wheezing/asthma (including lung function testing), and allergy (including skin prick testing) within the first 9 years of life; (5) neuropsychomotor assessments within the first 9 years of life (behavior, cognitive and psychomotor functions) (Table 2).

Main exposure assessments from prenatal until early school age periods focus on (1) metals (Pb, Cd, and Hg measured in blood, urine, or hair); (2) endocrine disruptors (phthalates and bisphenols in urine); (3) pesticides (organophosphates, pyrethroids, triazoles, and neocotinoids in urine); (4) air pollution (based on data from monitoring stations and biomarkers of exposure, such as PAHs in urine); (5) lifestyle-related and nutritional factors (smoking, micronutrients, and vitamins based on biomonitoring and questionnaires, while alcohol consumption, physical activity, new technologies, and psychosocial conditions are based on the latter); (6) socio-demographic determinants (based on questionnaires) (Table 2). DNA-methylation was also measured in cord blood using the Illumina Infinium Human Methylation450 BeadChip (Illumina Inc., San Diego, CA, USA). 

The laboratory analyses were performed on an ongoing basis and biobanking was created to store biological samples and made available for future analyses.

#### 3.1.4. Power/Sample Size Calculation

**Figure 2:**
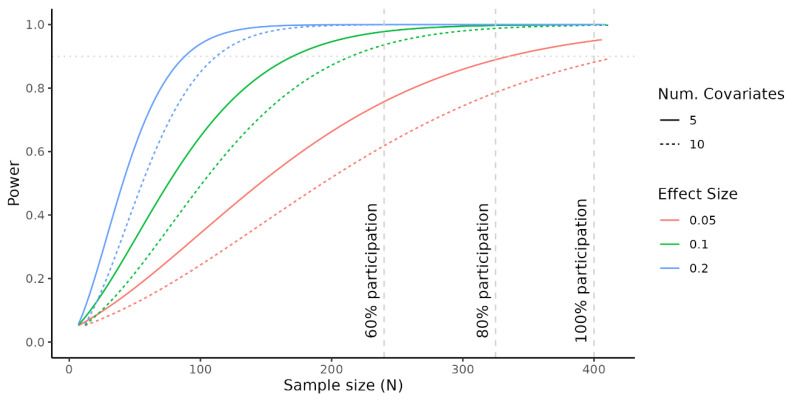
Power analysis calculations based on different assumptions: (i) participation rates of 100% (N = 400 adolescents), 80% (N = 320), and 60% (N = 240), represented by vertical grey-colored dashed lines; (ii) effect sizes of 0.05 (red line), 0.1 (green line), and 0.2 (blue line); (iii) number of covariates to be included in the regression models: 5 (continuous line) and 10 (dashed line). An optimal power of 0.9 is represented by an horizontal grey-colored dotted line. All these calculations are based on a significance level of alpha = 0.05.

Phase IV of the study aims at recruiting a total of 400 adolescents, for which the full spectrum of exposure and outcome assessment will be performed. Power analysis calculations for multiple linear regression have been performed based on three participation rates (100%, 80%, and 60%, corresponding to sample sizes of N = 400, N = 320, and N = 240 adolescents). In addition, different effect sizes (f^2^) have been set (0.05, 0.1, and 0.2, representing very small effects and hypothesizing R^2^ values between 5 and 15), as well as assuming a total of five or ten explanatory variables to be included in the model and a significance level of alpha = 0.05. The calculations have been conducted in R using the *pwr* package [57,58]. Figure 2 shows the results of the power analysis based on the aforementioned assumptions: different participation rates, effect sizes, and number of covariates to be included in the models. For effect sizes of 0.1 and 0.2, a power of 0.9 would be achieved, even with a 60% participation rate (Figure 2, green and blue lines). In the case of very small sizes (f^2^ = 0.05, red lines of Figure 2), a power of 0.9 would be accomplished when the participation rate would be at least 80% and a maximum of 5 explanatory variables would be used (red continuous line of Figure 2).

### 3.2. Phase IV of REPRO_PL–Adolescence Period

#### 3.2.1. Assessment of Exposure

The exposure assessment in adolescents will be based on questionnaires (filled in by themselves and/or by their parents), biomarkers of exposure (analyses of urine, blood, saliva, and hair samples), and personal monitoring tools (Table 2).

Lifestyle assessment based on questionnaire data. Adolescents will fill in a detailed questionnaire regarding risky behaviors (alcohol, tobacco, and e-cigarettes experimentation). Physical activity will be assessed based on the Polish version of Physical Activity Questionnaire (7-day recall used to assess general physical activity levels-https://journals.humankinetics.com/view/journals/pes/9/4/article-p342.xml, (accessed on 23 September 2022) and 7-day physical activity measurement using a smartwatch (wrist heart rate recorded by the wrist heart rate monitor—GENEActive). Diet will be evaluated by a 24 HR questionnaire filled in for three days (two questionnaires on a weekday and one on the weekend). A separate questionnaire will assess the use of mobile communication devices (mobiles and cordless phones, tablets, and laptops). Adolescents will provide information about the time spent using them (at school/work, at home, in public places and while commuting), the functions they employ and the time they use the devices in different positions.

Chemical exposure. A spot morning urine sample collected from the adolescents will be stored at NIOM laboratory and will be used for measurements of chemicals’ concentrations (metals, cotinine, PAH, phthalates, bisphenols and pesticides). Total mercury (THg) concentration will be determined by the Thermal Decomposition Amalgamation/Atomic Absorption Spectrometry method (TDA–AAS) using the Direct Mercury Analyzer (DMA-80 by Milestone, Spectro-Lab, Poland), whereas inductively coupled plasma mass spectrometry (ICP-DRC-MS) (Perkin Elmer, SCIEX, USA) will be used for the analysis of As, Cd and Pb. Cotinine level in the adolescents’ urine will be analyzed using LC-ESI-MS/MS, whereas PAH metabolites (1-hydroxypyrene, 3-, 4-, 9-hydroxyphenanthrene) will be analyzed using HPLC. The analysis of phthalates (21 metabolites), bisphenols (BPA, BPS, and BPF) and pesticides is also planned to be analyzed by liquid chromatography coupled to tandem mass spectrometry techniques. The levels of Pb and Cd will also be assessed in blood, whereas hair will be collected from adolescents to measure Hg.

Air pollution assessment will be performed based on the universal Kriging methodology where average concentrations of air pollutants (PM_2.5_, PM_10_, NO_2_ and SO_2_) from the entire country will be used and assigned to the residential addresses of the participants. Kriging is a geostatistical method of spatial modeling, in which apart from the values defined in the measurement points, the spatial variability in the phenomenon is also acknowledged, by the means of a regionalized variable. This variable has the properties of both random and deterministic variables, and its essential feature is the spatial correlation that characterizes each pair of points separated from each other by a certain distance. Apart from spatial correlation analysis, spatial estimation methods—which for places where no information is available, allow estimation of values based on measurements in adjacent points—are also used in Kriging. Other spatiotemporal models might be used, such as land use regression or hierarchical Bayesian spatiotemporal models, which are also based on monitoring stations and specific periods of interest. Air pollution measurements (PM_2.5_ and PM_10_ sampling in 24 h) using GilAir Plus Basic (Clearwater, FL, USA) personal aspirators are also planned in a subset of participants (N = 250 adolescents).

#### 3.2.2. Adolescents’ Health and Neuropsychomotor Development Assessment

A detailed questionnaire will be filled in by the mothers, covering information about their child’s health status, previous hospitalizations, medication use and allergy and asthma diagnosis (from doctor), based on ISAAC. The clinical examination will be performed by an allergologist during a scheduled visit. The following test/examinations/assessments will also be performed.

- Pulmonary function tests. Spirometry will be performed using a Master Screen unit (Erich Jaeger GmbhHochberg, Germany) according to the American Thoracic Society (ATS) and European Respiratory Society (ERS) guidelines. The results are expressed as percentages of the predicted values. Reversibility testing is performed after administration of salbutamol (400 μg). The percentage change from baseline in FEV1, pre-bronchodilatory FEV1, and PEF will be included in the analysis. The exhaled nitric oxide measurements will be performed according to ATS/ERS recommendations with a chemiluminescence analyzer (model 280i nitric oxide analyzer; Sievers, Boulder, CO, USA) and defined in parts per billion. The mean value of three successive, reproducible recordings of FENO will be retained for the statistical analysis.
- Skin prick testing (SPT) will be performed with the most common inhalant allergens: Dermatophagoides farinae, Dermatophagoides pteronyssinus, Alternaria, Cladosporium, cat dander, dog dander, mixed grass pollen, rye, birch, hazel, ribwort, alder, and mugwort together with a positive (histamine) and negative (glycerol) control (extracts from Allergopharma-Nexter, Reinbeg, Germany). A positive SPT reaction is defined as a mean weal diameter >3 mm in excess of the negative control.
- Weight and height assessment (as well as body composition) will be performed by a nurse using a height analyzer and TANITA-weight/body composition analyzer.
- Systolic and diastolic blood pressure will be measured twice, at least 5 min apart, in a resting position.
- During a separate morning visit, the blood will be collected and analyzed for thyroid stimulating hormone (TSH), triiodothyronine (FT3), thyroxine (FT4), lipid profile (cholesterol, low-density lipoprotein cholesterol (LDL), high-density lipoprotein cholesterol (HDL), and triglycerides (TG)), and glucose and insulin level.
- Faulty posture, persistent reflexes, balance, and gait parameters will be examined by a physiotherapist. The posture will be assessed using a Zebris system. Visual assessment is made on the frontal and posterior side in the frontal plane and in the sagittal plane on the right and left sides of the subject. The examination also includes tests assessing persistent reflexes that could affect changes in posture and balance. These are: the Asymmetrical Tonic Neck Reflex (ATNR), the Symmetrical Tonic Neck Reflex (STNR), and the Tonic Labyrinthine Reflex (TLR). The last element of the examination is the analysis of balance and gait parameters using MediPost based on inertial sensors—available at no cost for the current study. The static posturography protocol includes assessment of balance in a standing position on a stable surface with eyes open and closed, and on a foam surface with eyes open and closed.
- Sleep quality will be evaluated based on a modified version of the Sleep Disturbance Scales. It is designed to measure length and quality of sleep. The sleep quality is additionally verified by the 7-days smartwatch records.
- Adolescent neuropsychomotor assessment. The behavioral status will be assessed by the Strengths and Difficulties Questionnaire (SDQ), which is filled in by both the parent and the adolescent (https://www.sdqinfo.com, accessed on 23 September 2022). The SDQ is a widely used tool (frequently used in European birth cohorts) for the evaluation of the behavior, with a 25-item questionnaire consisting of four subscales measuring mental health problems (Conduct problems, Emotional symptoms, Peer relationship problems, and Hyperactivity/Inattention problems) and one sub-scale measuring strengths (Pro-social behavior). To assess intelligence and psychomotor abilities, the Intelligence and Development Scales for Children and Adolescents (IDS-2) is applied (https://www.practest.com.pl/ids-2-skale-inteligencji-i-rozwoju-dla-dzieci-i-mlodziezy, (accessed on 23 September 2022). The examination is performed by trained and certified psychologists. The IDS-2 is a tool designed for a thorough evaluation of skills and competences in persons aged 5–20. The battery includes 30 tests to examine cognitive skills (intelligence and executive functions) and competences (psychomotoric, socio-emotional, and school competences as well as work attitude). IDS-2 represent very high internal consistencies and stabilities for the IQ and factor scores, in addition to a >0.70 reliability of intelligence tests. High reliability of the general executive functions index as well as about a 0.80 reliability of tests scores have been achieved in the past.

### 3.3. Data Management

The questionnaire data from Phases I–III were collected in paper version. In Phase IV, mothers and adolescents have the possibility to fill in the electronic or the paper version questionnaires. The data from paper version will be entered into electronic databases by trained staff. From all questionnaires, five percent of them will be randomly double-checked by the research team to monitor the quality of the manual data entry process. The percentage of mistakes should not be exceeded by 2% (a similar percentage was obtained in Phases I–III). The exposure (results of laboratory analyses) and outcome data (related to adolescent’s health and neurodevelopment) will be entered into separate databases and will be linked to the questionnaire data by a unique identification (ID) number. Final databases will be checked by the study team. The ranges, distributions, means, standard deviations, outliers, and logical errors will be identified afterward. Data outliers and missing values will also be checked with the original forms. To answer specific research questions, each time database will be centrally created from different databases (the new database includes only variables needed for the specific analyses). All information in these datasets that enables identification of a particular participant, such as names or addresses, is excluded before distribution to the researchers. A protocol and plan for analysis as well as a data transfer agreement is required before data can be provided to the research team.

### 3.4. Ethics and Privacy Protection

All four phases of REPRO_PL were approved by the Bioethical Committee of the Nofer Institute of Occupational Medicine, Lodz, Poland (Decision No. 7/2007, 3/2008, 22/2014, 3/2021) and separate approval was also obtained for Phase III from the Medical Ethics Committee of the Medical University of Lodz (Decision No. RNN/388/17/KE). Written informed consents for the study are obtained from the subjects as a first step, before any study procedure is performed (as phase IV of the study involves adolescents at age 14 years, informed consent is to be signed by participants and separately by their parents or legal guardians/custodians). Detailed information sheets about the study have been given to the study participants and separately to their parents/legal guardians/custodians. This document describes the aims, methods, and implications of the research, the nature of participation, and any benefits, risks, or discomfort that might occur. It also includes information that participation is voluntary and anyone has the right to refuse to participate and to withdraw their participation, samples, data at any time without any consequences. The study is carried out according to existing guidance in ethics such as that laid down in the Universal Declaration on Bioethics and Human Rights adopted by UNESCO’s General Conference on 19 October 2005, the Council of Europe Convention for the Protection of Human Rights and Dignity of the Human Being, with regard to the Application of Biology and Medicine (1997) and as specified in the Helsinki Declaration (2000). Considering the prospective nature of the study, the research team obtained written informed consents signed within the previous phases of the study (Phases I–III) for the use of the data for future analyses (for scientific purposes focusing on the assessment of the impact of environmental and lifestyle-related factors on health and neurodevelopment) and for contact for follow-up visits. All the data and biological samples are only used for scientific purposes (analyses and publication of the results according to the project objectives). Personal data are protected according to current national and European regulations. The study will work according to strict rules of security and confidentiality according to the 1. Regulation (EU) 2016/679 of the European Parliament and of the Council of 27 April 2016 on the protection of natural persons with regard to the processing of personal data and on the free movement of such data, and repealing Directive 95/46/EC (General Data Protection Regulation) 2. The Act of 10 May 2018 on the protection of personal data (Journal of Laws of 2018, Item 1000). To protect the confidentiality of the participants and their information, unique identity codes (ID) are assigned and used on record forms and all the biological samples. The names and personal information of the participants (address, phone numbers, and e-mails) are kept separated from the body of the record forms and from the sample labels, and will only be accessible by the authorized research staff as well as by the research team involved in contacting and examining the participants. The key linking the identification numbers to personal data is stored in an encrypted electronic file protected by a password and stored in the secured electronic server, with limited access by the authorized research staff.

## 4. Strengths and Weaknesses of the REPRO_PL Cohort Study

The REPRO_PL cohort is conducted in central Europe, a location in which longitudinal studies covering a wide range of exposures and health effects are less frequently implemented. The evaluation of health effects is carried out using standardized and validated questionnaires and tools, which are the same as—or comparable to—those used in other birth cohorts. Moreover, analyses of biological samples are performed in accredited laboratories using up-to-date methodologies. This also provides a guarantee of reliable data and is the basis for inclusion in international analyses and collaborations. In REPRO_PL, most of the laboratory analyses are performed on an ongoing basis, while biobanking is also created.

The main limitation of the study is related to the REPRO_PL sample size, which may pose a challenge for certain analyses (i.e., rare exposure or outcomes, analysis considering genetic susceptibility, trajectories or exposome approaches). In those scenarios, combined analyses based on data from other existing cohorts might overcome the difficulties. The REPRO_PL cohort seems to be slightly over-represented by families with a high level of education or socioeconomic affluence. The weaknesses in estimating environmental exposure to contaminants need to be underlined (this can be related to the matrix and method selected for assessment, single sample collection, and selected cut-off). Finally, the losses of follow-up, which are common limitations of prospective studies, need to be pointed out. Major efforts are made to minimize losses of follow-up, by keeping the adolescents and their parents involved in the study. Several strategies have been implemented and are currently part of the study design. First, the research team has a wide range of contact information from the participants such as residential address, phone numbers, e-mail addresses, as well as close family contact details. This increases the chances of reaching a large proportion of potential study participants. Second, to increase the participation in the study, the day and time of the visits have been scheduled after each participant’s agreement. If a mother and the adolescent are not able to come and participate in the scheduled visit, a new appointment (convenient to them) is arranged. After the examination, all the participants receive the results, their interpretation, and some recommendations for improving their health status (i.e., skin prick and pulmonary functions tests, thyroid hormones, glucose, insulin, lipid profile levels, detailed description of offspring’s health by allergologists), neurobehavioral assessment (by psychologists), nutritional status (based on the 24 h dietary recall questionnaires, 24 HR), physical activity (based on the questionnaire data and wrist heart rate monitors), and anthropometric measurements and faulty posture (by physiotherapists). If needed, a new appointment with the general practitioner or a specialist is arranged for them. Finally, support for completing the questionnaires is offered, while the importance of study participation and completion of all their elements is explained in detail.

## 5. Collaboration

The REPRO_PL cohort is coordinated by a research team that includes epidemiologists, public health specialists, and toxicologists/biomonitoring experts from the Nofer Institute of Occupational Medicine (NIOM), always in close collaboration with the Department of Pediatrics and Allergy of the Medical University of Lodz. Various research analyses have already been performed and scientific papers were published as part of previous and ongoing European or worldwide collaboration projects. Given the nature of a prospective cohort study and the fact that research questions are not yet definitive but rather open for more developments, REPRO_PL creates a unique opportunity for further cross-cohort collaboration, in the form of joint analyses, scientific publications, and research projects.

## 6. Conclusions

The extension of the REPRO_PL cohort with examinations of adolescents at the age of 14 years old will provide a better understanding of the relationships between environmental and lifestyle-related factors and children’s health and neurodevelopment. It will also create a unique opportunity for cross-cohort collaborations within Europe, to ultimately strengthen the scientific base for further policies and interventions promoting health and wellbeing.

## Figures and Tables

**Table 1:** General characteristics of women and their children during phases I, II, and III of the REPRO_PL study.

	Prenatal Period (Phase I: T1–T3, Birth) *n* = 1266	Early Childhood (Phase II: 1 y/2 ys) *n* = 547	Early School Age (Phase III: 7–9 ys) *n* = 498
Mothers			
Age at child birth (years) mean ± SD	29.2 ± 4.2	29.6 ± 4.2	29.5 ± 4.0
Education (years of completed education) %			
≤9	2.3	2.2	2.1
10–12	27.6	29.6	30
>12	69.3	68.3	67.9
Pre-pregnancy BMI (kg/m^2^) mean ± SD	22.3 ± 3.6	22.6 ± 3.8	22.4 ± 3.7
Smoking (based on cotinine level in 1st trimester of pregnancy) %			
Yes	14.8	14.5	14
No	85.2	85.5	86
Alcohol consumption during pregnancy %			
Yes	7.8	7.2	8.9
No	92.2	92.8	91.1
Children			
Sex %			
Male	51.2	49.5	49
Female	48.8	50.5	51
Birth weight (grams) mean ± SD *	3367.5 ± 476.9	3337.0 ± 478.3	3477.2 ± 478.3
Gestational age (weeks) mean ± SD	39.2 ± 1.4	39.2 ± 1.4	39.3 ± 1.4

* *p*-value < 0.05.

**Table 2:** Exposures, outcomes, and covariates available within Polish Mother and Child Cohort (REPRO_PL).

	Prenatal Period (Phase I: T1–T3, Birth)	Early Childhood (Phase II: 1 y/2 ys)	Early School Age (Phase III: 7–9 ys)	Adolescent (Phase IV: 14 ys) Ongoing	Main Publications from REPRO_PL
**Exposure**					
Phthalates (21 metabolites)	Urine (T3)	Urine	Urine	*Urine*	[14,15,16,17,18,19,20]
Bisphenols (BPA, BPS, BPF)	-	-	Urine	*Urine*	[21]
Heavy Metals (Pb, Cd, Hg, As) ^1^	Maternal blood, Cord blood, Hair	-	*Urine*	*Urine//Blood/Hair*	[17,22,23,24,25,26]
Cotinine	Saliva (T1-T3)	Urine	Urine	*Urine*	[26,27,28]
Pesticides (metabolites of pyrethroids, organophosphates, triazoles, neocotinoids)			Urine	*Urine*	
Persistent Organic Pollutants				*Blood*	
Microelements and vitamins (Zn, Cu, Se, vit. A, E, D)	Maternal blood (T1-T3), Cord blood	-	-	-	[22,25,29,30,31,32,33,34,35,36]
Air pollution PAH (1-hydroxypyrene, 3-, 4-, 9-hydroxyphenanthrene) Kriging methodology Personal monitoring	Urine (T2) PM_10_, PM_2.5_ (T1-T3) -	Urine PM_10_, PM_2,5_ -	Urine PM_10_, PM_2,5_	*Urine* *PM_10_, PM_2,5,_, NO_2_, SO_2_* *GilAir personal aspirators*	[26,37,38] [39]
Lifestyle					
Diet	FFQ (T2),	Breastfeeding	24 HR (parent report)	*24 HR/FFQ* *(adolescent report)*	[33,40,41,42,43,44,45,46,47,48]
Alcohol consumption	Questionnaire (T1–T3)	-	-	*Questionnaire* *(adolescent report)*	[49]
Smoking (active/passive)	Questionnaire (T1–T3)	Questionnaire	Questionnaire (parent report)	*Questionnaire (tobacco/e-cigarettes)* *(adolescent report)*	[49,50]
Physical activity	Questionnaire (T1–T3)	-	Questionnaire (parent report)	*Questionnaire (PAQ), SW* *(adolescent report)*	[49,51]
New technologies	Questionnaire (T3)	-	Questionnaire (parent report)	*Questionnaire* *(adolescent report)*	
Psychosocial conditions	SWCQ, PSS, SRRS, APGAR (T2)	-	Mothers: PAS, EMQ (mother report)	*Questionnaire* *(adolescent report)*	[52,53]
**Outcomes**					
Child health		Questionnaire	Questionnaire	*Questionnaire*	
Birth outcomes	Medical records	-	-	*-*	[20,44,50,51]
Clinical examination	Neonatologist	Pediatrician	Pediatrician/allergologist	*Pediatrician/allergologist*	
Atopy	-	Questionnaire	Skin prick test	*Skin prick test*	[18,30,31,34,35,40,52,54,55,56]
Pulmonary function	-	-	Spirometry	*Spirometry*	[34,40]
Blood pressure	-	-	2 measurements	*2 measurements*	
Thyroid hormones (FT3, FT4, TSH)	-	-	-	*Blood*	
Glucose and insulin	-	-	-	*Blood*	
Lipid profile (cholesterol, LDL, HDL, TG)	-	-	-	*Blood*	
Puberty	-	-	-	*Questionnaire (parent/adolescent report)*	
Adiposity	-	Anthropometric measurements	Anthropometric measurements	*Anthropometric measurements*	[45]
Faulty posture	-	-	-	*Zebris system*	
Persistent reflexes	-	-	-	*ATNR, STNR, TLR*	
Balance and gait parameters	-	-	-	*MediPost based on inertial sensors*	
Sleeping pattern ^2^	-	-	-	*SDSC (parent/adolescent report)*	
Neurobehavioral outcomes					
Behavior	-	-	SDQ (parent report)	*SDQ (parent/adolescent report)*	[15,16,17,21,22,39,43]
Psychomotor and cognitive development	-	BSID-III	IDS	*IDS*	[15,16,17,21,22,24,25,26,29,32,49,53]
**Biobanking (−80 °C)**					
DNA	Cord blood	-	Buccal swabs	*Buccal swabs*	
Blood/Plasma	X (Plasma T1–T3, cord blood)		-	X	
Nasopharyngeal microbiome	-	-	-	X	
Urine	X (T2,T3)	X	X	X	
Saliva	X (T1–T3)		X	X	
Hair	X (T3)	-	-	X	
**Covariates**					
Personal and sociodemographic data	Questionnaire (T1–T3)	Questionnaire	Questionnaire *(parent report)*	*Questionnaire (parent/adolescent report)*	
Home environment	Questionnaire (T1–T3)	Questionnaire	Questionnaire *(parent report)*	*Questionnaire (parent report)*	
Family functioning	Questionnaire (T1–T3)	Questionnaire	Questionnaire *(parent report)*	*Questionnaire (parent report)*	

^1^ Metals: Cd, Pb—maternal blood (T2) and cord blood, Hg—maternal hair (T3), As—child urine at age 14 years; ^2^ Sleep quality is considered as exposure (cardiometabolic health) or outcome (new technologies use) in the analyses, SW—smartwatch (GENEActiv), T1—first trimester of pregnancy, T2—second trimester of pregnancy, T3—third trimester of pregnancy, FFQ—Food Frequency Questionnaire, 24 HR—24 h Dietary Recall Questionnaire, BSID III—Bayley Scale for Infant and Toddler Development, IDS—Intelligence and Development Scales, SDQ—Strength and Difficulties Questionnaire, ATNR—asymmetrical Tonic Neck Reflex, STNR—Symmetrical Tonic Neck Reflex, TLR—Tonic Labyrinthine Reflex, SDSC—sleep disturbance Scale for Children, PAQ—Physical Activity Questionnaire, SWCQ—Subjective Work Characteristics Questionnaire, PSS—Perceived Stress Scale, SRRS—Social Readjustment Rating Scale, APGAR Family Scale, PAS—Parental Attitudes Scale, EMQ—Eating Maturity Questionnaire (mothers). The planned measurements are shown in italics.

## Data Availability

Not applicable.
